# Surveillance Following Hepatitis B Surface Antigen Loss: An Issue Requiring Attention

**DOI:** 10.3390/pathogens14010008

**Published:** 2024-12-27

**Authors:** Shuai-Wen Huang, Hong Long, Jia-Quan Huang

**Affiliations:** 1 Department of General Practice, Tongji Hospital, Tongji Medical College, Huazhong University of Science and Technology, 1095 Jiefang Avenue, Wuhan 430030, China; longhong@hust.edu.cn; 2 Division of Nephrology, Tongji Hospital, Tongji Medical College, Huazhong University of Science and Technology, 1095 Jiefang Avenue, Wuhan 430030, China; 3 Department and Institute of Infectious Disease, Tongji Hospital, Tongji Medical College, Huazhong University of Science and Technology, 1095 Jiefang Avenue, Wuhan 430030, China; huangjiaquan21@aliyun.com; 4 Department of Nutrition, Tongji Hospital, Tongji Medical College, Huazhong University of Science and Technology, 1095 Jiefang Avenue, Wuhan 430030, China

**Keywords:** HBsAg loss, surveillance, HBsAg recurrence, HBV reactivation, HBV reinfection, cirrhosis, hepatocellular carcinoma

## Abstract

Due to the lack of agents that directly target covalently closed circular DNA and integrated HBV DNA in hepatocytes, achieving a complete cure for chronic hepatitis B (CHB) remains challenging. The latest guidelines recommend (hepatitis B surface antigen) HBsAg loss as the ideal treatment target for improving liver function, histopathology, and long-term prognosis. However, even after HBsAg loss, hepatitis B virus can persist, with a risk of recurrence, reactivation, cirrhosis, and hepatocellular carcinoma. Therefore, follow-up and surveillance are still necessary. With increasing treatment options available for achieving HBsAg loss in patients with CHB, developing effective surveillance strategies has become crucial. Recent studies on outcomes following HBsAg loss provide new insights for refining current surveillance strategies, though further improvement is needed through long-term observation and follow-up.

## 1. Introduction

Owing to the comprehensive implementation of mother-to-child hepatitis B virus (HBV) prevention and hepatitis B vaccine immunization programs, there has been a significant decline in the incidence of new HBV infections. However, it is important to note that a substantial global burden involving individuals infected with HBV remains [1,2,3,4,5,6,7]. Thus, HBV infection continues to pose a significant public health challenge worldwide.

The course of HBV infection is primarily determined by the interplay between the virus and host, with age at the time of infection being widely recognized as a critical factor influencing chronicity [7,8]. In adults, HBV infection typically resolves spontaneously, as marked by the clearance of serum HBV DNA and emergence of hepatitis B core antibodies (anti-HBc), leading to loss of HBsAg and often accompanied by seroconversion to anti-HBs (antibodies to hepatitis B surface antigen). Conversely, perinatal transmission is associated with a higher risk of chronic infection characterized by persistent positivity for both HBsAg and/or HBV DNA, which accounts for most cases in highly endemic regions [7].

The objective of treating chronic hepatitis B (CHB) is to effectively suppress HBV replication and ultimately reduce the incidence of complications such as cirrhosis and hepatocellular carcinoma (HCC) [9,10,11,12]. Nevertheless, due to the lack of agents that directly target covalently closed circular DNA (cccDNA) and integrated HBV DNA in hepatocytes, a complete cure for CHB remains challenging [10,12,13]. HBsAg loss has been linked to improved liver function, histopathology, and long-term prognosis, making it an ideal treatment goal recommended by recent guidelines for preventing and managing CHB [11,12]. It is generally believed that only a small proportion of individuals with chronic HBV infection can achieve a spontaneous loss of HBsAg and attain a state similar to recovery from acute infection characterized by sustained and stable host immune control over HBV.

**Figure 1:**
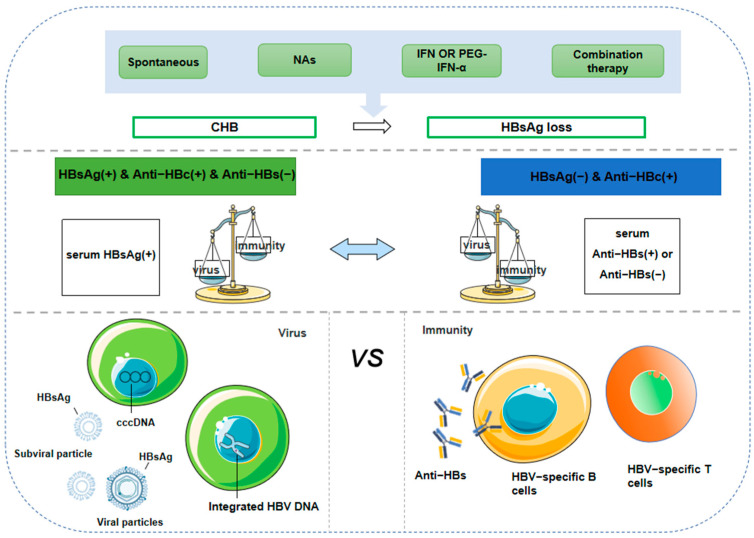
HBsAg loss implies stable immune control of HBV.

The loss of HBsAg is a complex immune process that involves cell-mediated immune responses, antibody production, the formation of immune memory, and overcoming immune evasion [14]. In some patients, the immune system is able to successfully clear HBV by recognizing and eliminating infected hepatocytes or neutralizing the virus, leading to the loss of HBsAg. However, this process is not always smooth. In CHB patients, immune tolerance may prevent the virus from being fully cleared [15]. Immune evasion, viral mutations, and the intensity of the immune response all play crucial roles in this process [14,16,17]. In most cases, HBsAg loss indicates stable immune control over HBV(Figure 1), which subsequently promotes the clearance of residual virus, including cccDNA and integrated HBV DNA.

Currently approved drugs for CHB mainly consist of nucleos(t)ide analogues (NA) and interferon drugs. Although NA are an effective treatment for CHB, achieving HBsAg loss with NA drug therapy is rare and may require long-term therapy [18]. Extended courses of treatment with NA, adherence to medication, and safety remain significant challenges in the management of CHB treatment. A limited course of NA therapy has been proposed as an alternative to long-term therapy with the potential to increase HBsAg loss [19,20], yet virological rebound and recurrence after discontinuation are almost universal [21]. Thus, achieving safe withdrawal from NA remains controversial. In 2005, pegylated interferon alpha (PEG-IFN-α) was approved for treating patients with CHB [22]. A limited course of PEG-IFNα-based therapy (sequential or in combination with NA) resulted in increased HBsAg loss. With progress achieved through clinical studies, rational optimization of PEG-IFNα-based treatment for select groups has significantly increased the loss of HBsAg [23,24,25,26,27,28]. Despite these promising results for future Peg-IFNα therapy, it is poorly tolerated due to the subcutaneous administration requirement and potential adverse reactions.

The current focus of drug development for hepatitis B encompasses two main aspects: targeting the life cycle of HBV and modulating the host immune system. Direct antiviral agents that specifically target the HBV life cycle include entry inhibitors [29], capsid inhibitors [30], small interfering RNAs [31], antisense oligonucleotides [32], and HBsAg inhibitors [33]. Indirect antiviral agents that modulate human immunity consist of therapeutic vaccines [34], monoclonal antibodies [35], and immune checkpoint inhibitors [36], among others. Several phase II/III clinical trials are being performed to simultaneously address both the HBV life cycle and host immune modulation. Some of these studies have demonstrated promising research progress and hold the potential to provide additional options for achieving HBsAg loss. For further details on the progress in the development of new hepatitis B drugs, some recently published review articles may be referred to [37,38,39].

Compared to sustained virological replication inhibition, HBsAg loss has been shown to effectively reduce the risk of hepatocellular carcinoma in patients with CHB [40,41,42,43]. However, due to the lack of effective means for complete eradication of cccDNA and integration into host genomic DNA, the hepatitis B virus can persist even after acute self-limited hepatitis B recovery or CHB infection, leading to HBsAg loss [13,44,45,46]. Consequently, seroreversion of HBsAg and reactivation of HBV can occur spontaneously or be triggered under specific conditions [13,47]. Furthermore, although the risk of hepatocellular carcinoma is significantly reduced following HBsAg loss, it remains a risk [48,49,50,51].

Clinical and animal experiments provide evidence indicating that traces of HBV DNA may persist in the liver for years or decades after HBsAg loss in acute or CHB and are associated with hepatic histological abnormalities [44,46,52,53,54,55,56]. Although HBsAg loss is an ideal endpoint for CHB infection, large-scale population cohort studies have confirmed that, after adjusting for confounding factors, the incidence of hepatocellular cancer (HCC) (HR: 7.95, 95% CI: 3.50–18.04) [57] and overall mortality risk [58] are higher in individuals after the resolution of an HBV infection than in those without previous HBV infection. Overall, the persistence of HBV leads to a continued risk of HBsAg recurrence, reactivation, cirrhosis, and hepatocellular carcinoma subsequent to HBsAg loss. Consequently, post-HBsAg loss follow-up and surveillance remain imperative.

## 2. HBsAg Recurrence or HBV Reactivation After HBsAg Loss

Loss of HBsAg generally indicates sustained immune control over HBV, but serological recurrence or reactivation may occur due to persistent viral presence and an imbalance between host immunity and viral replication. Biologics (such as CD20 monoclonal antibody), immunosuppressants (such as corticosteroids), chemotherapy agents, and anti-hepatitis C drugs can accelerate seroreversion or HBV reactivation in an immunosuppressive state [55,59,60,61,62,63,64,65]. Additionally, spontaneous HBV reactivation can occur without any specific trigger [66]. Nonimmunosuppressive states with seroreversion of HBsAg loss and hepatitis B virus reactivation following immunosuppression are described separately below.

### 2.1. HBsAg Recurrence in a Nonimmunosuppressive State

A recent large-scale community survey found that the 10-year cumulative seroreversion rate of HBsAg in the population after HBsAg loss was approximately 1.9%, with a higher rate observed in individuals who were anti-HBs negative than in those who were anti-HBs positive (10-year cumulative seroreversion rate: 2.9% vs. 1.4%). Notably, individuals who had previously received hepatitis B treatment showed a surprisingly high incidence of seroreversion, at 40.45%, with a higher incidence in the anti-HBs-negative group than in the anti-HBs-positive group (the 10-year cumulative incidence of seroreversion: 50.6% vs. 33.4%). Advanced age, surgical history, surface antibody negativity, HCV antibody positivity, and increased BMI and AFP levels are independent risk factors for seroreversion [67]. A large-scale inpatient study conducted in Hong Kong that excluded groups likely to be co-immunosuppressed (e.g., co-HCV, immunosuppressive, and biologics) reported that the incidence of seroreversion after spontaneous HBsAg loss during CHB was 2.1% and that the incidence of seroreversion after NA-induced HBsAg loss was 2.9% [68]. Although two studies based on community and hospital populations reported different conclusions regarding incidence and risk factors, it is generally accepted that seroreversion of HBsAg in the absence of an immunosuppressive state is transient with no obvious clinical symptoms and does not lead to true disease reactivation [67,68]. Smaller studies have suggested varying rates of seroreversion after NA-induced HBsAg loss, ranging from 3.9% (median follow-up of 26 months) [69] to 7.2% (mean follow-up of six years) [70]. Studies have also shown that liver cirrhosis is a risk factor for serologic reversal of HBsAg in patients with NA-induced HBsAg loss, though withdrawal of NA does not seem to be associated with serologic recurrence [69].

In recent years, sequential or combined administration of interferon and nucleos(t)ide analogue (NA) drugs has significantly enhanced the likelihood of HBsAg loss in individuals with CHB. The persistence of interferon-induced HBsAg loss has become a focal point of research. One study with a median follow-up period of 3.3 years demonstrated an approximate cumulative incidence rate of serologic recurrence after IFN-induced HBsAg loss of 9.6%, and low levels of hepatitis B surface antibody (anti-HBs) were identified as a significant risk factor for serologic recurrence [71]. Another prospective cohort study lasting 48 weeks reported a cumulative incidence rate of serologic recurrence after IFN-induced HBsAg loss of approximately 12.79%, with higher levels of anti-HBs along with post-HBsAg loss interferon consolidation therapy being predictive factors for sustained functional cure [72]. Additionally, lower levels of hepatitis B core antibody (anti-HBc) may be associated with an increased risk of serological recurrence [73]. According to recent literature published by Professor Ning Qin, short-term incidence rates for seroreversion following IFN-induced HBsAg loss are approximately 41.2%. Lower levels of hepatitis B core-related antigen (HBcrAg) and higher levels of anti-HBs reflect reduced intrahepatic cccDNA levels, more stable proportions of HBV-specific cytotoxic T lymphocytes, and sustained follicular T-helper and B-cell immune responses, which serve as effective indicators for predicting persistent surface antigen loss [74].

Table 1 summarizes the incidence rates and potential predictors for seroreversion after HBsAg loss. The reported incidence of serological HBsAg reversal varies among previous studies, as influenced by factors such as the study population, mode of infection, treatment duration, hepatitis B epidemic environment, and laboratory precision. In most cases, persistence is observed following HBsAg loss. Even if transient seroreversion occurs without obvious clinical symptoms in the absence of immunosuppression, these individual patients still have strong response rates to IFN or NA-based therapy [75].

Certain factors and models for predicting durable functional cures have been proposed, but there are inconsistencies in results between studies, and external validation remains weak due to the limited number of relapse cases with HBsAg loss. Further research is needed to identify high-risk groups for recurrence after HBsAg loss and to determine appropriate clinical treatment for those who experience relapse.

### 2.2. HBV Reactivation After HBsAg Loss in Immunosuppressed States

As there is currently no effective eradication therapy for HBV, a significant proportion of the general population remains at risk of HBV reactivation, particularly those who are either infected with or have been exposed to HBV but have achieved HBsAg loss. The potential consequences of such reactivation are especially concerning when these individuals undergo cancer chemotherapy, immunosuppression, or biological therapies for conditions such as rheumatism, malignancy, inflammatory bowel disease, skin disease, or solid organ or bone marrow transplantation [83]. Reactivation may occur due to decreased host immune control over the virus or increased viral replication resulting from immunosuppression [13]. Under such circumstances, serious adverse events, including liver failure and death, can ensue.

In a systematic meta-analysis, populations negative for HBsAg but positive for anti-HBc exhibited an approximate HBV reactivation rate of 6.5% when treated with immunosuppressants [84]. Among these populations, individuals with hematologic diseases (10.9%) and those receiving rituximab (9.7%) were found to have the highest risk of reactivation; baseline detectable HBV DNA and anti-HBs negativity were identified as significant risk factors for reactivation. Under severe immunosuppressive conditions, HBV reactivation can be fatal, as evidenced by the eventual death of 14.9% of patients experiencing such reactivation in the meta-analysis; however, the exact relationship between the cause of death and HBV reactivation remains inconclusive [84].

Guidelines from the American Gastroenterological Association recommend anti-viral agents for preventing viral reactivation in intermediate- and high-risk populations requiring long-term immunologic therapy against hepatitis B [85]. This recommendation is particularly relevant with high doses or prolonged courses of glucocorticoids and anthracycline derivatives (e.g., doxorubicin, epirubicin) in patients who are negative for both HBsAg and anti-HBs but positive for anti-HBc [86,87,88]. Although some of the literature suggests that individuals with detectable levels of anti-HBs have a relatively low risk of HBV reactivation, long-term immunologic therapy is not recommended by American Gastroenterological Association guidelines because a subset of anti-HBs-positive patients are still at risk; therefore, early prophylaxis with antivirals is strongly advised based on substantial clinical evidence and other guidelines to prevent serious adverse events [85,89,90,91].

Reactivation of HBV can occur in individuals who are negative for HBsAg, particularly those receiving anti-CD20 antibodies or undergoing hematopoietic stem cell transplantation. However, as newer targeted biological therapies emerge, it is important to understand which therapies may also predispose towards HBV reactivation. Table 2 summarizes immunosuppressive drugs or therapies that may be associated with HBV reactivation following HBsAg loss. It should be noted that these data and information may rapidly change due to the complex clinical application background, and risk ratings may need timely adjustment based on new clinical evidence.

A meticulous approach is needed to manage individuals with a history of HBV infection who are HBsAg-negative but anti-HBc-positive, especially in regions with high HBV prevalence. Although the risk of HBV reactivation is a concern for these individuals, it may not be appropriate to apply the same treatment to all HBV-infected individuals and HBsAg-positive patients without clear evidence to support it; treatment should be personalized. However, when facing extremely high risks of reactivation and severe adverse outcomes, prophylactic antiviral treatment should indeed be considered.

Additionally, aging individuals may experience HBV reactivation even without traditional triggers [66], but the effects of immunosenescence in such cases remain poorly understood and require further research. Few studies have assessed the risk of reactivation and treatment monitoring of HBV in elderly populations; thus, whether additional attention is needed for this group remains unclear.

## 3. Hepatitis B Virus Reinfection After HBsAg Loss

The issue of HBV reinfection remains a concern in the field of liver transplantation, particularly in areas with high hepatitis B prevalence and limited donor sources [84]. In certain cases, the selection of HBsAg-positive or anti-HBc-positive donors may be necessary, despite the infection potential. Distinguishing between reinfection and reactivation can be challenging in practice, and both are significant risks for transplant recipients due to persistence of the virus and long-term immunosuppressive therapy.

There have been reports of switching of HBsAg−/anti-HBc+/anti-HBs+ cases to HBsAg+ cases at 3.5 months after HBsAg+ donor liver transplantation due to the decrease in anti-HBs titer. Interestingly, however, HBsAg is cleared at 18 months after liver transplantation in patients with high anti-HBs titers [101]. This seems to suggest that this distribution of liver transplantation may be feasible, and although HBsAg+ donor liver transplantation may lead to reinfection, there is still a chance that individuals who have previously achieved HBsAg loss will regain relatively stable immunologic control of HBV. For anti-HBc+ donor liver transplantation, the rate of reinfection in HBsAg−/anti-HBc+/anti-HBs+ recipients is reportedly significantly lower than that of HBsAg−/anti-HBc−/anti-HBs+ recipients or HBsAg−/anti-HBc−/anti-HBs− recipients (1.4 versus 9.4 percent versus 13 percent), which may be associated with immune memory preservation after anti-HBs and previous hepatitis B infection [102].

Although reinfection with hepatitis B is generally less likely to occur after HBsAg loss due to the immune memory of previous HBV infection, the occurrence of reinfection after liver transplantation suggests that reinfection may in theory still occur after HBsAg loss; thus, reducing hepatitis B virus exposure may still bring benefits to the population achieving HBsAg loss.

## 4. Cirrhosis Combined with HBsAg Loss

Prior to the onset of cirrhosis, HBsAg loss is associated with a lower risk of developing cirrhosis [103]. However, particular attention should be given to those who experience HBsAg loss while already having cirrhosis, as they still have a high incidence of HCC even after HBsAg loss.

According to the literature, the proportion of patients with cirrhosis at the time of HBsAg loss is relatively high in the total population with HBsAg loss. A large cohort study from the Hong Kong Medical Authority showed that 6.3% (451/7124) of HBsAg-cleared HBV-infected patients had cirrhosis and that 3.5% (247/7124) had decompensated cirrhosis [104]. In a multicenter cohort study in South Korea, this figure was as high as 39.9% [69].

The incidence of cirrhosis after HBsAg clearance is relatively low [105], though the risk is still present [48,51,76], and some scholars believe that previous HBV infection may be the underlying cause of some cases of cryptogenic cirrhosis [106]. Early HBsAg loss is associated with less severe liver fibrosis [107,108,109]. Cirrhosis may occur during the HBV carrier phase of CHB infection and may also be associated with latent hepatitis B infection. A recent study based on liver biopsy proposed important risk factors for cirrhosis and tumor development in patients with nonalcoholic fatty liver disease previously infected with hepatitis B [110]. Similarly, some studies have confirmed that prior hepatitis B infection is associated with an increased risk of cirrhosis and liver cancer in patients with hepatitis C [111,112]. A study from Korea estimated the prevalence of cirrhosis after CHB clearance to be approximately 39.9%. Such higher prevalence may be related to the timing of cirrhosis, and the study did not make a strict distinction between cirrhosis occurring during the HBsAg carrier period of CHB infection and cirrhosis occurring after HBsAg clearance. The incidence of newly diagnosed cirrhosis following HBsAg loss is relatively low [50,76,107]; however, there remains a dearth of large-scale prospective cohort studies in this area. Table 3 presents a summary of the prevalence of cirrhosis in populations infected with HBV and demonstrating a loss of HBsAg.

## 5. Occurrence and Prediction of HCC After HBsAg Loss

The risk of hepatocellular carcinoma is significantly reduced after HBsAg loss, but numerous studies have shown that this risk still exists [113,115]. A large registry study of women of childbearing age suggests that even with the loss of HBsAg, the risk of HCC is much higher than that in the population without hepatitis B infection (HR = 7.95, 95% CI: 3.50–18.04) [57]. Based on the results of several recently published systematic meta-analyses, the incidence of HCC following HBsAg loss is approximately 1.86 to 2.29% [116,117,118], though a recent study from South Korea suggested that the incidence of HCC after HBsAg loss can be as high as 4.9% [119]. Such variation may be due to the prevalence of hepatitis B in the population and country involved.

The mechanisms underlying the development of hepatocarcinoma may involve the integration of HBV DNA into the host genome, and these genetic alterations can lead to the clonal selection of noncancerous hepatocytes with survival advantages, resulting in the development of liver tumors through the random insertion of HBV DNA into host hepatocytes [119,120]. It has also been suggested that the lower risk of hepatocellular carcinoma following HBsAg loss may be associated with incomplete HBsAg loss [121].

The development of HCC following HBsAg loss appears to be strongly associated with sex and age [42]. Studies suggest that the risk of HCC is higher in men and in those older than 50 years [42]. Cirrhosis and the development of diabetes mellitus are also risk factors for HCC [105]. Enhanced liver fibrosis testing may also play a role in the risk stratification of HCC [122].

Most models for predicting HCC are based on HBsAg-positive patients. However, only the CU-HCC and PAGE-B models have been cross-validated in HBsAg-negative populations [40,113]. Recently, Hyun Yang et al. proposed a predictive model for tumorigenesis in HBsAg-negative populations, but this model lacks effective external validation [119].

## 6. Surveillance Strategies Following HBsAg Loss

Continued surveillance is important to monitor for reactivation, relapse, reinfection, cirrhosis, or HCC, particularly in those with a history of chronic infection. For high-risk groups, regular testing for HBsAg, HBV DNA, anti-HBs, liver function, liver ultrasound, and AFP is still necessary to monitor viral response, liver health, and immune status. Emerging non-invasive biomarkers, such as HBV RNA [123], HBcrAg [74], HBsAg isoform quantification [124], high-sensitivity quantitative HBsAg [125], and HBV core antibodies [126], have gained attention for reflecting intrahepatic viral activity and assessing the likelihood of achieving partial or complete functional cure. However, most of these are still in the early research stages. The optimal frequency and methods for monitoring outcomes after HBsAg loss have yet to be determined, and their cost-effectiveness remains unknown. This underscores the need for further research.

The results from high-sensitivity HBsAg assays have prompted researchers to re-evaluate findings from previous studies. With improvements in measurement methods, it has recently become possible to detect trace amounts of HBsAg. In long-term observational studies, for example, patients previously judged as HBsAg-negative due to older, less sensitive methods may be considered HBsAg-positive with recent, more sensitive tests. In their review, Wang, Z.L. et al. discuss the potential of using high-sensitivity HBsAg assays as an indicator of complete cure [125]. However, even with high-sensitivity assays, cases of HBV reactivation have still been reported [127]. Research on long-term outcomes following HBsAg clearance using high-sensitivity tests remains limited. The optimal precision for detecting HBsAg remains unclear, and the health economic cost-effectiveness of these tests is yet to be established.

Indeed, current research suggests that HBsAg loss alone may not be sufficient to diagnose a complete cure for hepatitis B, as it does not necessarily indicate the elimination of all HBV-infected cells or the prevention of HBV reactivation. Although studies have explored various biomarkers and approaches, including high-sensitivity HBsAg assays and other viral markers, none have yet provided a universally accepted standard for diagnosing a complete cure. This gap highlights an ongoing need for further research and consensus in the field.

To design an implementation framework for monitoring after HBsAg loss, with a particular focus on its application in developing countries, we recommend adopting a prospective, longitudinal observational study design to track chronic hepatitis B patients who have cleared HBsAg. Future research should focus on evaluating the impact of long-term monitoring on patient health outcomes and exploring the feasibility and implementation barriers of monitoring protocols in countries with varying economic levels. Monitoring will include regular testing for HBV DNA, liver function, and imaging exams. The study will cover different monitoring periods, including early, intermediate, and long-term follow-ups, ensuring a comprehensive assessment of health changes after HBsAg clearance. Given the resource limitations in developing countries, the framework emphasizes delivering convenient monitoring services through primary healthcare settings, using cost-effective and simple testing methods, such as HBV DNA rapid tests, liver elastography, and ultrasound. The study will also place a strong emphasis on training healthcare providers and educating patients to improve adherence to long-term monitoring. Furthermore, collaborations with governments and international organizations will be explored to address economic burdens and ensure the sustainability of these monitoring programs in resource-limited settings. The results of this study will not only contribute to improving the management of hepatitis B patients’ health but also provide valuable insights for the formulation of global hepatitis B prevention and control policies.

## 7. Future Directions and Challenges

HBsAg loss is associated with improved liver function, histopathological improvement, and long-term prognosis and is the ideal treatment target recommended by the latest guidelines for the prevention and treatment of CHB. However, given the persistent challenges in completely eradicating the hepatitis B virus, it appears that individuals previously infected with the hepatitis B virus but achieving HBsAg loss require ongoing monitoring to facilitate timely personalized prevention and intervention measures for adverse outcomes. Nevertheless, current surveillance strategies following HBsAg loss necessitate further refinement and improvement. Future studies with long-term observation and follow-up of this population should be conducted to provide additional clinical evidence and guidance for developing effective surveillance strategies. In addition, further research is needed to identify suitable non-invasive biomarkers as surrogate indicators for sustained or complete cure of hepatitis B.

## Figures and Tables

**Table 1:** Incidence and predictors of seroreversion of HBsAg after HBsAg loss.

Author	Way to Achieve HBsAg Loss	Follow-Up (Years)	Incidence of Seroreversion	Predictors of Durable Functional Cure
ML Yeh [67]	Unknown	4.2 ^1^	1.1% (84/7630)	See text
T.C.Yip [68]	Spontaneous		2.1% (75/3563)	-
T.C.Yip [68]	NAs	-	2.9% (14/475)	-
M.A.Kim [69]	NAs	2.2 ^1^	3.6% (10/276)	-
G.Kim [70]	NAs	6 ^1^	7.2% (8/110)	-
Y. Wu [71]	IFN or combined with NA therapy	3.3 ^1^	7.5% (18/238)	Anti-HBs ^3^
M.H. Li [72]	IFN/NAs add on IFN	1	12.7% (22/172)	IFN therapy > 12 weeks ^4^; Anti-HBs ^3^
Y. Wu [73]	IFN or combined with NA therapy	2	21.9% (16/73)	Anti-HBc ^5^
D. Huang [74]	NAs followed by IFN	0.5	41.6% (15/36)	HBcrAg < 4 logU/mL & antiHBs > 2 log IU/L levels ^6^
X. Lin [75]	IFN or combined with NA therapy	4.2 ^1^	8.9% (32/358)	-
H.Chi [76]	NAs	1.6 ^1^	3.7% (2/54)	-
MF. Han [77]	NAs followed by IFN	1	14.2% (1/7)	-
X. LIN [75]	IFN	4.3 ^1^	8.9% (32/358)	Anti-HBs ^3^; Anti-HBc ^5^
G.Teuber [78]	NAs combined with IFN	3 ^1^	2.1% (3/143)	-
M. Li [79]	IFN or combined with NA therapy	2	8.2% (19/231)	IFN therapy > 12 weeks ^4^;
N. Gao [80]	IFN	1	13.5% (30/222)	Anti-HBs ^3^
A.S.Alawad [81]	Spontaneous/NAs/IFN	9.6 ^2^	4.6% (3/65)	-
A.S.Lok [82]	NAs/IFN/combined	2 ^1^	18.1% (10/55)	-

^1^ Median follow-up time; ^2^ mean follow-up duration; ^3^ anti-HBs ≥ 100 IU/L; ^4^ IFN consolidation treatment is longer than 12 weeks; ^5^ those with higher anti-HBc titers may achieve more durable functional cure; ^6^ treatment endpoint HbcrAg < 4 log10 U/mL and anti-HBs > 2 log10 IU/L may result in a more durable functional cure.

**Table 2:** Immunosuppressive drugs or therapies associated with HBV reactivation after HBsAg loss.

Category	Drug/Therapy	Potential Clinical Application Scenarios	HBV Reactivation
CD20 monoclonal antibody	Rituximab	Hematological tumors, rheumatoid arthritis, idiopathic thrombocytopenic purpura, multiple sclerosis	9.7% [84]
Tumor chemotherapeutic drug	Doxorubicin, Epirubicin	Solid and hematological malignancies	11.3% [61]
Tumor chemotherapeutic drug	Platinum compound	Solid tumor	3.0% [92]
TNF-α inhibitors	Infliximab	Inflammatory bowel disease, psoriasis, ankylosing spondylitis, rheumatoid arthritis	0.0–0.4% [62,93]
Calcineurin inhibitors	Cyclosporin, Tacrolimus	Solid organ transplantation, rheumatoid arthritis, psoriasis, aplastic anemia	10.0% [94,95]
Chemokine inhibitors	Moglizumab	Refractory adult T-cell leukemia/lymphoma	12.5% [96]
Janus kinase inhibitors	Brectinib	Rheumatoid arthritis	14.0% [94,97]
Cytokine inhibitor	Tocilizumab	Rheumatoid arthritis	2.0% [94,98]
CAR-T therapy	CAR-T therapy	Refractory/recurrent diffuse large B-cell lymphoma	3.3% [94,99]
Cortisol hormone	Cortisol hormone	A variety of allergic diseases, widely used	3.0% [94,100]
Immune checkpoint inhibitors	PD-1 inhibitors	Tumor immunotherapy	0.0–1% [94]

**Table 3:** Prevalence of cirrhosis in populations infected with HBV and exhibiting HBsAg loss.

Author	Way to HBsAg Loss	Cirrhosis Before/After HBsAg Loss	Criterion	Number of Patients	Prevalence
T.C.Yip [104]	Spontaneous/NAs	-	ICD code	7124	6.3%
M.A.Kim [69]	NAs	-	Imaging evaluation	276	39.9%
Y.Park [113]	Spontaneous/antiviral therapy	-	Imaging evaluation and FIB-4	1200	13.8%
Y.C.Chen [114]	Spontaneous/NAs	-	Pathology/Imaging evaluation	422	10.2%
G.A. Kim [40]	Spontaneous/NAs	-	Imaging evaluation	829	11.8%
Y. Arase [107]	-	After HBsAg loss	Pathology	164	0.0%
H.Chi [76]	NAs	After HBsAg loss	Imaging evaluation	70	0.0%
Y.C.Chen [50]	-	After HBsAg loss	Imaging evaluation	189	1.6%

## References

[1] Schmit N., Nayagam S., Thursz M.R., Hallett T.B. (2021). The global burden of chronic hepatitis b virus infection: Comparison of country-level prevalence estimates from four research groups. Int. J. Epidemiol..

[2] Hsu Y.-C., Huang D.Q., Nguyen M.H. (2023). Global burden of hepatitis b virus: Current status, missed opportunities and a call for action. Nat. Rev. Gastroenterol. Hepatol..

[3] Papastergiou V., Lombardi R., MacDonald D., Tsochatzis E.A. (2015). Global epidemiology of hepatitis b virus (hbv) infection. Curr. Hepatol. Rep..

[4] Cortesi P.A., Fornari C., Conti S., Antonazzo I.C., Ferrara P., Ahmed A., Andrei C.L., Andrei T., Artamonov A.A., Banach M. (2023). Hepatitis b and c in europe: An update from the global burden of disease study 2019. Lancet Public Health.

[5] Cui F., Blach S., Mingiedi C.M., Gonzalez M.A., Alaama A.S., Mozalevskis A., Séguy N., Rewari B.B., Chan P.-L., Le L.-V. (2023). Global reporting of progress towards elimination of hepatitis b and hepatitis c. Lancet Gastroenterol. Hepatol..

[6] Tan M., Bhadoria A.S., Cui F., Tan A., Van Holten J., Easterbrook P., Ford N., Han Q., Lu Y., Bulterys M. (2021). Estimating the proportion of people with chronic hepatitis b virus infection eligible for hepatitis b antiviral treatment worldwide: A systematic review and meta-analysis. Lancet Gastroenterol. Hepatol..

[7] Tang L.S., Covert E., Wilson E., Kottilil S. (2018). Chronic hepatitis b infection: A review. JAMA.

[8] Iannacone M., Guidotti L.G. (2022). Immunobiology and pathogenesis of hepatitis B virus infection. Nat. Rev. Immunol..

[9] Kao J.H., Hu T.H., Jia J., Kurosaki M., Lim Y.S., Lin H.C., Sinn D.H., Tanaka Y., Wai-Sun Wong V., Yuen M.F. (2020). East Asia expert opinion on treatment initiation for chronic hepatitis B. Aliment. Pharmacol. Ther..

[10] Cornberg M., Lok A.S.F., Terrault N.A., Zoulim F., Berg T., Brunetto M.R., Buchholz S., Buti M., Chan H.L., The 2019 EASL-AASLD HBV Treatment Endpoints Conference Faculty (2020). Guidance for design and endpoints of clinical trials in chronic hepatitis B—Report from the 2019 EASL-AASLD HBV Treatment Endpoints Conference. Hepatology.

[11] Terrault N.A., Lok A.S., McMahon B.J., Chang K.M., Hwang J.P., Jonas M.M., Brown R.S., Bzowej N.H., Wong J.B. (2018). Update on prevention, diagnosis, and treatment of chronic hepatitis B: AASLD 2018 hepatitis B guidance. Hepatology.

[12] (2017). EASL 2017 Clinical Practice Guidelines on the management of hepatitis B virus infection. J. Hepatol..

[13] Shi Y., Zheng M. (2020). Hepatitis B virus persistence and reactivation. BMJ (Clin. Res. Ed.).

[14] Ferrari C. (2015). HBV and the immune response. Liver Int..

[15] Bertoletti A., Kennedy P.T. (2015). The immune tolerant phase of chronic HBV infection: New perspectives on an old concept. Cell. Mol. Immunol..

[16] Kuipery A., Gehring A.J., Isogawa M. (2020). Mechanisms of HBV immune evasion. Antivir. Res..

[17] Sinha P., Thio C.L., Balagopal A. (2024). Intracellular Host Restriction of Hepatitis B Virus Replication. Viruses.

[18] Chevaliez S., Hézode C., Bahrami S., Grare M., Pawlotsky J.M. (2013). Long-term hepatitis B surface antigen (HBsAg) kinetics during nucleoside/nucleotide analogue therapy: Finite treatment duration unlikely. J. Hepatol..

[19] Hirode G., Choi H.S.J., Chen C.H., Su T.H., Seto W.K., Van Hees S., Papatheodoridi M., Lens S., Wong G., Brakenhoff S.M. (2022). Off-therapy response after nucleos(t)ide analogue withdrawal in patients with chronic hepatitis b: An international, multicenter, multiethnic cohort (RETRACT-B study). Gastroenterology.

[20] Wong G.L., Chan H.L., Yuen B.W., Tse Y.K., Luk H.W., Yip T.C., Hui V.W., Liang L.Y., Lee H.W., Lui G.C. (2020). The safety of stopping nucleos(t)ide analogue treatment in patients with HBeAg-negative chronic hepatitis B. Liver Int. Off. J. Int. Assoc. Study Liver.

[21] Lampertico P., Berg T. (2018). Less can be more: A finite treatment approach for HBeAg-negative chronic hepatitis B. Hepatology.

[22] Schinazi R.F., Ehteshami M., Bassit L., Asselah T. (2018). Towards HBV curative therapies. Liver Int. Off. J. Int. Assoc. Study Liver.

[23] Marcellin P., Ahn S., Chuang W.L., Hui A., Tabak F., Mehta R., Petersen J., Lee C.M., Ma X., Caruntu F. (2016). Predictors of response to tenofovir disoproxil fumarate plus peginterferon alfa-2a combination therapy for chronic hepatitis B. Aliment. Pharmacol. Ther..

[24] Ning Q., Han M., Sun Y., Jiang J., Tan D., Hou J., Tang H., Sheng J., Zhao M. (2014). Switching from entecavir to PegIFN alfa-2a in patients with HBeAg-positive chronic hepatitis B: A randomised open-label trial (OSST trial). J. Hepatol..

[25] Hagiwara S., Nishida N., Watanabe T., Ida H., Sakurai T., Ueshima K., Takita M., Komeda Y., Nishijima N., Osaki Y. (2018). Sustained antiviral effects and clearance of hepatitis surface antigen after combination therapy with entecavir and pegylated interferon in chronic hepatitis B. Antivir. Ther..

[26] Bourliere M., Rabiega P., Ganne-Carrie N., Serfaty L., Marcellin P., Barthe Y., Thabut D., Guyader D., Hezode C., Picon M. (2017). Effect on HBs antigen clearance of addition of pegylated interferon alfa-2a to nucleos (t) ide analogue therapy versus nucleos (t) ide analogue therapy alone in patients with HBe antigen-negative chronic hepatitis B and sustained undetectable plasma hepatitis B virus DNA: A randomised, controlled, open-label trial. Lancet Gastroenterol. Hepatol..

[27] Lim S.G., Yang W.L., Chang J.P.E., Ngu J., Tan Y.-L.J., Ahmed T., Dan Y.Y., Lee Y.M., Lee G.H., Tan P.S. (2019). Switch or add-on peginterferon to chronic hepatitis b patients already on nucleos (t) ide analogue therapy (SWAP study): Final results. Hepatology.

[28] Bazinet M., Pântea V., Placinta G., Moscalu I., Cebotarescu V., Cojuhari L., Jimbei P., Iarovoi L., Smesnoi V., Musteata T. (2020). Safety and efficacy of 48 weeks REP 2139 or REP 2165, tenofovir disoproxil, and pegylated interferon Alfa-2a in patients with chronic HBV infection naïve to nucleos(t)ide therapy. Gastroenterology.

[29] Kirstgen M., Müller S.F., Lowjaga K.A.A.T., Goldmann N., Lehmann F., Alakurtti S., Yli-Kauhaluoma J., Baringhaus K.-H., Krieg R., Glebe D. (2021). Identification of novel HBV/HDV entry inhibitors by pharmacophore-and QSAR-guided virtual screening. Viruses.

[30] Yuen M.F., Berliba E., Sukeepaisarnjaroen W., Ahn S.H., Tanwandee T., Lim Y.S., Kim Y.J., Poovorawan K., Tangkijvanich P., Schwabe C. (2022). Safety, pharmacokinetics, and antiviral activity of the capsid inhibitor AB-506 from Phase 1 studies in healthy subjects and those with hepatitis B. Hepatol. Commun..

[31] Hou J., Zhang W., Xie Q., Hua R., Tang H., Morano Amado L.E., Yang S.-S., Peng C.-Y., Su W.-W., Chuang W.-L. (2024). Xalnesiran with or without an immunomodulator in chronic hepatitis B. N. Engl. J. Med..

[32] Yuen M.-F., Heo J., Jang J.-W., Yoon J.-H., Kweon Y.-O., Park S.-J., Tami Y., You S., Yates P., Tao Y. (2021). Safety, tolerability and antiviral activity of the antisense oligonucleotide bepirovirsen in patients with chronic hepatitis B: A phase 2 randomized controlled trial. Nat. Med..

[33] Chauhan R., Li Q., Woodson M.E., Gasonoo M., Meyers M.J., Tavis J.E. (2021). Efficient inhibition of hepatitis B virus (HBV) replication and cccDNA formation by HBV ribonuclease H inhibitors during infection. Antimicrob. Agents Chemother..

[34] Zoulim F., Fournier C., Habersetzer F., Sprinzl M., Pol S., Coffin C.S., Leroy V., Ma M., Wedemeyer H., Lohse A.W. (2020). Safety and immunogenicity of the therapeutic vaccine TG1050 in chronic hepatitis B patients: A phase 1b placebo-controlled trial. Hum. Vaccines Immunother..

[35] Yuen M.-F., Lim Y.-S., Yoon K.T., Lim T.-H., Heo J., Tangkijvanich P., Tak W.Y., Thanawala V., Cloutier D., Mao S. (2024). VIR-2218 (elebsiran) plus pegylated interferon-alfa-2a in participants with chronic hepatitis B virus infection: A phase 2 study. Lancet Gastroenterol. Hepatol..

[36] Bunse T., Kosinska A.D., Michler T., Protzer U. (2022). PD-L1 silencing in liver using siRNAs enhances efficacy of therapeutic vaccination for chronic hepatitis b. Biomolecules.

[37] Jeng W.-J., Lok A.S. (2023). What will it take to cure hepatitis B?. Hepatol. Commun..

[38] Ogunnaike M., Das S., Raut S.S., Sultana A., Nayan M.U., Ganesan M., Edagwa B.J., Osna N.A., Poluektova L.Y. (2023). Chronic hepatitis B infection: New approaches towards cure. Biomolecules.

[39] Lee H.W., Lee J.S., Ahn S.H. (2020). Hepatitis B virus cure: Targets and future therapies. Int. J. Mol. Sci..

[40] Kim G.-A., Lee H.C., Kim M.-J., Ha Y., Park E.J., An J., Lee D., Shim J.H., Kim K.M., Lim Y.-S. (2015). Incidence of hepatocellular carcinoma after HBsAg seroclearance in chronic hepatitis B patients: A need for surveillance. J. Hepatol..

[41] Kaur S.P., Talat A., Karimi-Sari H., Grees A., Chen H.W., Lau D.T., Catana A.M. (2022). Hepatocellular carcinoma in hepatitis B virus-infected patients and the role of hepatitis B surface antigen (HBsAg). J. Clin. Med..

[42] Yip T.C., Chan H.L., Wong V.W., Tse Y.K., Lam K.L., Wong G.L. (2017). Impact of age and gender on risk of hepatocellular carcinoma after hepatitis B surface antigen seroclearance. J. Hepatol..

[43] Song C., Zhu J., Ge Z., Yu C., Tian T., Wang H., Han J., Shen H., Dai J., Lu J. (2019). Spontaneous seroclearance of hepatitis B surface antigen and risk of hepatocellular carcinoma. Clin. Gastroenterol. Hepatol. Off. Clin. Pract. J. Am. Gastroenterol. Assoc..

[44] Rehermann B., Ferrari C., Pasquinelli C., Chisari F.V. (1996). The hepatitis B virus persists for decades after patients’ recovery from acute viral hepatitis despite active maintenance of a cytotoxic T–lymphocyte response. Nat. Med..

[45] Michalak T.I., Pasquinelli C., Guilhot S., Chisari F.V. (1994). Hepatitis B virus persistence after recovery from acute viral hepatitis. J. Clin. Investig..

[46] Yuki N., Nagaoka T., Yamashiro M., Mochizuki K., Kaneko A., Yamamoto K., Omura M., Hikiji K., Kato M. (2003). Long-term histologic and virologic outcomes of acute self-limited hepatitis B. Hepatology.

[47] Hoofnagle J.H. (2009). Reactivation of hepatitis B. Hepatology.

[48] Yuen M.F., Wong D.K.H., Fung J., Ip P., But D., Hung I., Lau K., Yuen J.C.H., Lai C.L. (2008). HBsAg Seroclearance in chronic hepatitis B in Asian patients: Replicative level and risk of hepatocellular carcinoma. Gastroenterology.

[49] Huo T.I., Wu J.C., Lee P.C., Chau G.Y., Lui W.Y., Tsay S.H., Ting L.T., Chang F.Y., Lee S.D. (1998). Sero-clearance of hepatitis B surface antigen in chronic carriers does not necessarily imply a good prognosis. Hepatology.

[50] Chen Y.C., Sheen I.S., Chu C.M., Liaw Y.F. (2002). Prognosis following spontaneous HBsAg seroclearance in chronic hepatitis B patients with or without concurrent infection. Gastroenterology.

[51] Kim J.H., Lee Y.S., Lee H.J., Yoon E., Jung Y.K., Jong E.S., Lee B.J., Seo Y.S., Yim H.J., Yeon J.E. (2011). HBsAg seroclearance in chronic hepatitis B: Implications for hepatocellular carcinoma. J. Clin. Gastroenterol..

[52] Liang T.J., Blum H.E., Wands J.R. (1990). Characterization and biological properties of a hepatitis B virus isolated from a patient without hepatitis B virus serologic markers. Hepatology.

[53] Michalak T.I., Pardoe I.U., Coffin C.S., Churchill N.D., Freake D.S., Smith P., Trelegan C.L. (1999). Occult lifelong persistence of infectious hepadnavirus and residual liver inflammation in woodchucks convalescent from acute viral hepatitis. Hepatology.

[54] Bläckberg J., Kidd-Ljunggren K. (2000). Occult hepatitis B virus after acute self-limited infection persisting for 30 years without sequence variation. J. Hepatol..

[55] Werle-Lapostolle B., Bowden S., Locarnini S., Wursthorn K., Petersen J., Lau G., Trepo C., Marcellin P., Goodman Z., Delaney W.E.t. (2004). Persistence of cccDNA during the natural history of chronic hepatitis B and decline during adefovir dipivoxil therapy. Gastroenterology.

[56] Gao N., Guan G., Xu G., Wu H., Xie C., Mo Z., Deng H., Xiao S., Deng Z., Peng L. (2023). Integrated HBV DNA and cccDNA maintain transcriptional activity in intrahepatic HBsAg-positive patients with functional cure following PEG-IFN-based therapy. Aliment. Pharmacol. Ther..

[57] Fwu C.W., Chien Y.C., Kirk G.D., Nelson K.E., You S.L., Kuo H.S., Feinleib M., Chen C.J. (2009). Hepatitis B virus infection and hepatocellular carcinoma among parous Taiwanese women: Nationwide cohort study. J. Natl. Cancer Inst..

[58] Jinjuvadia R., Liangpunsakul S., Antaki F. (2014). Past exposure to hepatitis B: A risk factor for increase in mortality?. J. Clin. Gastroenterol..

[59] Lau G.K., Yiu H.H., Fong D.Y., Cheng H.C., Au W.Y., Lai L.S., Cheung M., Zhang H.Y., Lie A., Ngan R. (2003). Early is superior to deferred preemptive lamivudine therapy for hepatitis B patients undergoing chemotherapy. Gastroenterology.

[60] Shibolet O., Ilan Y., Gillis S., Hubert A., Shouval D., Safadi R. (2002). Lamivudine therapy for prevention of immunosuppressive-induced hepatitis B virus reactivation in hepatitis B surface antigen carriers. Blood.

[61] Hsu C., Tsou H.H., Lin S.J., Wang M.C., Yao M., Hwang W.L., Kao W.Y., Chiu C.F., Lin S.F., Lin J. (2014). Chemotherapy-induced hepatitis B reactivation in lymphoma patients with resolved HBV infection: A prospective study. Hepatology.

[62] Pauly M.P., Tucker L.Y., Szpakowski J.L., Ready J.B., Baer D., Hwang J., Lok A.S. (2018). Incidence of hepatitis B virus reactivation and hepatotoxicity in patients receiving long-term treatment with tumor necrosis factor antagonists. Clin. Gastroenterol. Hepatol. Off. Clin. Pract. J. Am. Gastroenterol. Assoc..

[63] Cheng A.L., Hsiung C.A., Su I.J., Chen P.J., Chang M.C., Tsao C.J., Kao W.Y., Uen W.C., Hsu C.H., Tien H.F. (2003). Steroid-free chemotherapy decreases risk of hepatitis B virus (HBV) reactivation in HBV-carriers with lymphoma. Hepatology.

[64] Mücke M.M., Backus L.I., Mücke V.T., Coppola N., Preda C.M., Yeh M.L., Tang L.S.Y., Belperio P.S., Wilson E.M., Yu M.L. (2018). Hepatitis B virus reactivation during direct-acting antiviral therapy for hepatitis C: A systematic review and meta-analysis. Lancet Gastroenterol. Hepatol..

[65] Hwang J.P., Lok A.S. (2014). Management of patients with hepatitis B who require immunosuppressive therapy. Nat. Rev. Gastroenterol. Hepatol..

[66] Kamitsukasa H., Iri M., Tanaka A., Nagashima S., Takahashi M., Nishizawa T., Okamoto H. (2015). Spontaneous reactivation of hepatitis B virus (HBV) infection in patients with resolved or occult HBV infection. J. Med. Virol..

[67] Yeh M.L., Liang P.C., Huang C.I., Hsieh M.H., Lin Y.H., Jang T.Y., Wei Y.J., Hsu P.Y., Hsu C.T., Wang C.W. (2021). Seroreversion of hepatitis B surface antigen among subjects with resolved hepatitis B virus infection: A community-based cohort study. J. Gastroenterol. Hepatol..

[68] Yip T.C., Wong G.L., Wong V.W., Tse Y.K., Lui G.C., Lam K.L., Chan H.L. (2017). Durability of hepatitis B surface antigen seroclearance in untreated and nucleos(t)ide analogue-treated patients. J. Hepatol..

[69] Kim M.A., Kim S.U., Sinn D.H., Jang J.W., Lim Y.S., Ahn S.H., Shim J.J., Seo Y.S., Baek Y.H., Kim S.G. (2020). Discontinuation of nucleos(t)ide analogues is not associated with a higher risk of HBsAg seroreversion after antiviral-induced HBsAg seroclearance: A nationwide multicentre study. Gut.

[70] Kim G.A., Lim Y.S., An J., Lee D., Shim J.H., Kim K.M., Lee H.C., Chung Y.H., Lee Y.S., Suh D.J. (2014). HBsAg seroclearance after nucleoside analogue therapy in patients with chronic hepatitis B: Clinical outcomes and durability. Gut.

[71] Wu Y., Liu Y., Lu J., Cao Z., Jin Y., Ma L., Geng N., Ren S., Zheng Y., Shen C. (2020). Durability of interferon-induced hepatitis B surface antigen seroclearance. Clin. Gastroenterol. Hepatol. Off. Clin. Pract. J. Am. Gastroenterol. Assoc..

[72] Li M.H., Yi W., Zhang L., Lu Y., Lu H.H., Shen G., Wu S.L., Hao H.X., Gao Y.J., Chang M. (2019). Predictors of sustained functional cure in hepatitis B envelope antigen-negative patients achieving hepatitis B surface antigen seroclearance with interferon-alpha-based therapy. J. Viral Hepat..

[73] Wu Y., Wang X., Lin X., Shen C., Chen X. (2021). Quantitative of serum hepatitis B core antibody is a potential predictor of recurrence after interferon-induced hepatitis B surface antigen clearance. J. Microbiol. Immunol. Infect. (Wei Mian Yu Gan Ran Za Zhi).

[74] Huang D., Wu D., Wang P., Wang Y.L., Yuan W., Hu D.Q., Hu J.J., Wang Y.Q., Tao R., Xiao F. (2022). End-of-treatment HBcrAg and HBsAb levels identify durable functional cure after Peg-IFN-based therapy in patients with CHB. J. Hepatol..

[75] Lin X., Song A., Lu J., Zheng S., Hu Z., Ma L., Cao Z., Li H., Zheng Y., Ren S. (2022). Study on the retreatment, outcome, and potential predictors of recurrence in patients with recurrence of hepatitis B after functional cure. Front. Immunol..

[76] Chi H., Wong D., Peng J., Cao J., Van Hees S., Vanwolleghem T., Qi X., Chen L., Feld J.J., de Knegt R.J. (2017). Durability of response after hepatitis B surface antigen seroclearance during nucleos(t)ide analogue treatment in a multiethnic cohort of chronic hepatitis B patients: Results after treatment cessation. Clin. Infect. Dis. Off. Publ. Infect. Dis. Soc. Am..

[77] Han M., Jiang J., Hou J., Tan D., Sun Y., Zhao M., Ning Q. (2016). Sustained immune control in HBeAg-positive patients who switched from entecavir therapy to pegylated interferon-α2a: 1 year follow-up of the OSST study. Antivir. Ther..

[78] Teuber G., Sprinzl M., Hueppe D., Heyne R., Hofmann W.P., Cornberg M., Arne S., Petersen J. (2018). Durability of HBsAg loss during or after antiviral treatment in a predominantly middle European collective. J. Hepatol..

[79] Li M., Sun F., Bi X., Lin Y., Yang L., Lu Y., Zhang L., Wan G., Yi W., Zhao L. (2022). Consolidation treatment needed for sustained HBsAg-negative response induced by interferon-alpha in HBeAg positive chronic hepatitis B patients. Virol. Sin..

[80] Gao N., Yu H., Zhang J., Mo Z., Chu J., Xie C., Peng L., Gao Z. (2022). Role of hepatitis B surface antibody in seroreversion of hepatitis B surface antigen in patients achieving hepatitis B surface antigen loss with pegylated interferon-based therapy. J. Viral Hepat..

[81] Alawad A.S., Auh S., Suarez D., Ghany M.G. (2020). Durability of spontaneous and treatment-related loss of hepatitis B s antigen. Clin. Gastroenterol. Hepatol..

[82] Lok A.S., Zoulim F., Dusheiko G., Chan H.L.Y., Buti M., Ghany M.G., Gaggar A., Yang J.C., Wu G., Flaherty J.F. (2020). Durability of hepatitis B surface antigen loss with nucleotide analogue and peginterferon therapy in patients with chronic hepatitis B. Hepatol. Commun..

[83] Loomba R., Liang T.J. (2017). Hepatitis B reactivation associated with immune suppressive and biological modifier therapies: Current concepts, management strategies, and future directions. Gastroenterology.

[84] Cholongitas E., Haidich A.-B., Apostolidou-Kiouti F., Chalevas P., Papatheodoridis G.V. (2018). Hepatitis B virus reactivation in HBsAg-negative, anti-HBc-positive patients receiving immunosuppressive therapy: A systematic review. Ann. Gastroenterol..

[85] Reddy K.R., Beavers K.L., Hammond S.P., Lim J.K., Falck-Ytter Y.T. (2015). American gastroenterological association institute guideline on the prevention and treatment of hepatitis B virus reactivation during immunosuppressive drug therapy. Gastroenterology.

[86] Paul S., Dickstein A., Saxena A., Terrin N., Viveiros K., Balk E.M., Wong J.B. (2017). Role of surface antibody in hepatitis B reactivation in patients with resolved infection and hematologic malignancy: A meta-analysis. Hepatology.

[87] Jun C.H., Kim B.S., Oak C.Y., Lee D.H., Cho E., Cho S.B., Choi S.K., Park C.H., Joo Y.E., Lee J.J. (2017). HBV reactivation risk factors in patients with chronic HBV infection with low replicative state and resolved HBV infection undergoing hematopoietic stem cell transplantation in Korea. Hepatol. Int..

[88] Nishida T., Matsubara T., Yakushijin T., Inada M. (2019). Prediction and clinical implications of HBV reactivation in lymphoma patients with resolved HBV infection: Focus on anti-HBs and anti-HBc antibody titers. Hepatol. Int..

[89] Cao W., Wei J., Wang N., Xu H., Xiao M., Huang L., Cao Y., Li C., Xiao Y., Gu C. (2020). Entecavir prophylaxis for hepatitis B virus reactivation in patients with CAR T-cell therapy. Blood.

[90] Katz L.H., Fraser A., Gafter-Gvili A., Leibovici L., Tur-Kaspa R. (2008). Lamivudine prevents reactivation of hepatitis B and reduces mortality in immunosuppressed patients: Systematic review and meta-analysis. J. Viral Hepat..

[91] Loomba R., Rowley A., Wesley R., Liang T.J., Hoofnagle J.H., Pucino F., Csako G. (2008). Systematic review: The effect of preventive lamivudine on hepatitis B reactivation during chemotherapy. Ann. Intern. Med..

[92] Paul S., Saxena A., Terrin N., Viveiros K., Balk E.M., Wong J.B. (2016). Hepatitis B virus reactivation and prophylaxis during solid tumor chemotherapy: A systematic review and meta-analysis. Ann. Intern. Med..

[93] Fidan S., Capkın E., Arıca D.A., Durak S., Okatan I.E. (2021). Risk of hepatitis B reactivation in patients receiving anti-tumor necrosis factor-α therapy. Int. J. Rheum. Dis..

[94] Papatheodoridis G.V., Lekakis V., Voulgaris T., Lampertico P., Berg T., Chan H.L.Y., Kao J.H., Terrault N., Lok A.S., Reddy K.R. (2022). Hepatitis B virus reactivation associated with new classes of immunosuppressants and immunomodulators: A systematic review, meta-analysis, and expert opinion. J. Hepatol..

[95] Mikulska M., Nicolini L., Signori A., Rivoli G., Del Bono V., Raiola A.M., Di Grazia C., Dominietto A., Varaldo R., Ghiso A. (2014). Hepatitis B reactivation in HBsAg-negative/HBcAb-positive allogeneic haematopoietic stem cell transplant recipients: Risk factors and outcome. Clin. Microbiol. Infect. Off. Publ. Eur. Soc. Clin. Microbiol. Infect. Dis..

[96] Totani H., Kusumoto S., Ishida T., Masuda A., Yoshida T., Ito A., Ri M., Komatsu H., Murakami S., Mizokami M. (2015). Reactivation of hepatitis B virus (HBV) infection in adult T-cell leukemia-lymphoma patients with resolved HBV infection following systemic chemotherapy. Int. J. Hematol..

[97] Harigai M., Winthrop K., Takeuchi T., Hsieh T.Y., Chen Y.M., Smolen J.S., Burmester G., Walls C., Wu W.S., Dickson C. (2020). Evaluation of hepatitis B virus in clinical trials of baricitinib in rheumatoid arthritis. RMD Open.

[98] Rodríguez-Tajes S., Miralpeix A., Costa J., López-Suñé E., Laguno M., Pocurull A., Lens S., Mariño Z., Forns X. (2021). Low risk of hepatitis B reactivation in patients with severe COVID-19 who receive immunosuppressive therapy. J. Viral Hepat..

[99] Cui R., Lyu C., Li Q., Jiang Y., Mou N., Yang Z., Liu X., Deng Q., Li L. (2021). Humanized anti-CD19 chimeric antigen receptor-T cell therapy is safe and effective in lymphoma and leukemia patients with chronic and resolved hepatitis B virus infection. Hematol. Oncol..

[100] Wong G.L., Wong V.W., Yuen B.W., Tse Y.K., Yip T.C., Luk H.W., Lui G.C., Chan H.L. (2020). Risk of hepatitis B surface antigen seroreversion after corticosteroid treatment in patients with previous hepatitis B virus exposure. J. Hepatol..

[101] Loggi E., Bihl F., Chisholm J.V., Biselli M., Bontadini A., Vitale G., Ercolani G., Grazi G.L., Pinna A.D., Bernardi M. (2009). Anti-HBs re-seroconversion after liver transplantation in a patient with past HBV infection receiving a HBsAg positive graft. J. Hepatol..

[102] Russo F.P., Viganò M., Stock P., Ferrarese A., Pugliese N., Burra P., Aghemo A. (2022). HBV-positive and HIV-positive organs in transplantation: A clinical guide for the hepatologist. J. Hepatol..

[103] Moucari R., Korevaar A., Lada O., Martinot-Peignoux M., Boyer N., Mackiewicz V., Dauvergne A., Cardoso A.C., Asselah T., Nicolas-Chanoine M.H. (2009). High rates of HBsAg seroconversion in HBeAg-positive chronic hepatitis B patients responding to interferon: A long-term follow-up study. J. Hepatol..

[104] Yip T.C., Wong V.W., Tse Y.K., Liang L.Y., Hui V.W., Zhang X., Li G.L., Lui G.C., Chan H.L., Wong G.L. (2021). Similarly low risk of hepatocellular carcinoma after either spontaneous or nucleos(t)ide analogue-induced hepatitis B surface antigen loss. Aliment. Pharmacol. Ther..

[105] Vittal A., Sharma D., Hu A., Majeed N.A., Terry N., Auh S., Ghany M.G. (2022). Systematic review with meta-analysis: The impact of functional cure on clinical outcomes in patients with chronic hepatitis B. Aliment. Pharmacol. Ther..

[106] Morgan T.R., Redeker A.G., Yamada S., Ashcavai M. (1986). HBsAg clearance in chronic active hepatitis B. A possible cause of cryptogenic cirrhosis. Dig. Dis. Sci..

[107] Arase Y., Ikeda K., Suzuki F., Suzuki Y., Saitoh S., Kobayashi M., Akuta N., Someya T., Hosaka T., Sezaki H. (2006). Long-term outcome after hepatitis B surface antigen seroclearance in patients with chronic hepatitis B. Am. J. Med..

[108] Lauret E., González-Diéguez M.L., Rodríguez M., González M., Melón S., Rodrigo L., Rodríguez M. (2015). Long-term outcome in Caucasian patients with chronic hepatitis B virus infection after HBsAg seroclearance. Liver Int. Off. J. Int. Assoc. Study Liver.

[109] Lim T.H., Gane E., Moyes C., Borman B., Cunningham C. (2016). HBsAg loss in a New Zealand community study with 28-year follow-up: Rates, predictors and long-term outcomes. Hepatol. Int..

[110] Chan T.T., Chan W.K., Wong G.L., Chan A.W., Nik Mustapha N.R., Chan S.L., Chong C.C., Mahadeva S., Shu S.S., Lai P.B. (2020). Positive hepatitis B core antibody is associated with cirrhosis and hepatocellular carcinoma in nonalcoholic fatty liver disease. Am. J. Gastroenterol..

[111] Squadrito G., Cacciola I., Alibrandi A., Pollicino T., Raimondo G. (2013). Impact of occult hepatitis B virus infection on the outcome of chronic hepatitis C. J. Hepatol..

[112] Matsuoka S., Nirei K., Tamura A., Nakamura H., Matsumura H., Oshiro S., Arakawa Y., Yamagami H., Tanaka N., Moriyama M. (2008). Influence of occult hepatitis B virus coinfection on the incidence of fibrosis and hepatocellular carcinoma in chronic hepatitis C. Intervirology.

[113] Park Y., Lee J.H., Sinn D.H., Park J.Y., Kim M.A., Kim Y.J., Yoon J.H., Kim D.Y., Ahn S.H., Kang W. (2021). Risk and risk score performance of hepatocellular carcinoma development in patients with hepatitis B surface antigen seroclearance. Clin. Transl. Gastroenterol..

[114] Chen Y.C., Jeng W.J., Chien R.N., Chu C.M., Liaw Y.F. (2016). Clinical outcomes after spontaneous and nucleos(t)ide analogue-treated HBsAg seroclearance in chronic HBV infection. Aliment. Pharmacol. Ther..

[115] Pollicino T., Squadrito G., Cerenzia G., Cacciola I., Raffa G., Craxi A., Farinati F., Missale G., Smedile A., Tiribelli C. (2004). Hepatitis B virus maintains its pro-oncogenic properties in the case of occult HBV infection. Gastroenterology.

[116] Liu F., Wang X.W., Chen L., Hu P., Ren H., Hu H.D. (2016). Systematic review with meta-analysis: Development of hepatocellular carcinoma in chronic hepatitis B patients with hepatitis B surface antigen seroclearance. Aliment. Pharmacol. Ther..

[117] Kuang X.J., Jia R.R., Huo R.R., Yu J.J., Wang J.J., Xiang B.D., Li L.Q., Peng Z., Zhong J.H. (2018). Systematic review of risk factors of hepatocellular carcinoma after hepatitis B surface antigen seroclearance. J. Viral Hepat..

[118] Song A., Wang X., Lu J., Jin Y., Ma L., Hu Z., Zheng Y., Shen C., Chen X. (2021). Durability of hepatitis B surface antigen seroclearance and subsequent risk for hepatocellular carcinoma: A meta-analysis. J. Viral Hepat..

[119] Yang H., Bae S.H., Nam H., Lee H.L., Lee S.W., Yoo S.H., Song M.J., Kwon J.H., Nam S.W., Choi J.Y. (2022). A risk prediction model for hepatocellular carcinoma after hepatitis B surface antigen seroclearance. J. Hepatol..

[120] Jang J.W., Kim J.S., Kim H.S., Tak K.Y., Nam H., Sung P.S., Bae S.H., Choi J.Y., Yoon S.K., Roberts L.R. (2021). Persistence of intrahepatic hepatitis B virus DNA integration in patients developing hepatocellular carcinoma after hepatitis B surface antigen seroclearance. Clin. Mol. Hepatol..

[121] Suzuki F., Hosaka T., Imaizumi M., Kobayashi M., Ohue C., Suzuki Y., Fujiyama S., Kawamura Y., Sezaki H., Akuta N. (2021). Potential of ultra-highly sensitive immunoassays for hepatitis B surface and core-related antigens in patients with or without development of hepatocellular carcinoma after hepatitis B surface antigen seroclearance. Hepatol. Res. Off. J. Jpn. Soc. Hepatol..

[122] Mak L.Y., Cheung K.S., Hui R.W., Wong D.K., Fung J., Yuen M.F., Seto W.K. (2022). Enhanced liver fibrosis score stratifies hepatocellular carcinoma risk in patients with hepatitis B surface antigen seroclearance. Clin. Infect. Dis. Off. Publ. Infect. Dis. Soc. Am..

[123] Kasianchuk N., Dobrowolska K., Harkava S., Bretcan A., Zarębska-Michaluk D., Jaroszewicz J., Flisiak R., Rzymski P. (2023). Gene-editing and RNA interference in treating hepatitis B: A review. Viruses.

[124] Lazarevic I., Banko A., Miljanovic D., Cupic M. (2024). Hepatitis B surface antigen isoforms: Their clinical implications, utilisation in diagnosis, prevention and new antiviral strategies. Pathogens.

[125] Wang Z.L., Zheng J.R., Yang R.F., Huang L.X., Chen H.S., Feng B. (2023). An ideal hallmark closest to complete cure of chronic hepatitis B patients: High-sensitivity quantitative HBsAg loss. J. Clin. Transl. Hepatol..

[126] Lazarevic I., Banko A., Miljanovic D., Cupic M. (2023). Clinical utility of quantitative hbv core antibodies for solving diagnostic dilemmas. Viruses.

[127] Inoue T., Kusumoto S., Iio E., Ogawa S., Suzuki T., Yagi S., Kaneko A., Matsuura K., Aoyagi K., Tanaka Y. (2021). Clinical efficacy of a novel, high-sensitivity HBcrAg assay in the management of chronic hepatitis B and HBV reactivation. J. Hepatol..

